# 1p36 deletion syndrome: Review and mapping with further characterization of the phenotype, a new cohort of 86 patients

**DOI:** 10.1002/ajmg.a.63041

**Published:** 2022-11-11

**Authors:** Clémence Jacquin, Emilie Landais, Céline Poirsier, Alexandra Afenjar, Ahmad Akhavi, Nathalie Bednarek, Caroline Bénech, Adeline Bonnard, Damien Bosquet, Lydie Burglen, Patrick Callier, Sandra Chantot‐Bastaraud, Christine Coubes, Charles Coutton, Bruno Delobel, Margaux Descharmes, Jean‐Michel Dupont, Vincent Gatinois, Nicolas Gruchy, Sarah Guterman, Abdelkader Heddar, Lucas Herissant, Delphine Heron, Bertrand Isidor, Pauline Jaeger, Guillaume Jouret, Boris Keren, Paul Kuentz, Cedric Le Caignec, Jonathan Levy, Nathalie Lopez, Zoe Manssens, Dominique Martin‐Coignard, Isabelle Marey, Cyril Mignot, Chantal Missirian, Céline Pebrel‐Richard, Lucile Pinson, Jacques Puechberty, Sylvia Redon, Damien Sanlaville, Marta Spodenkiewicz, Anne‐Claude Tabet, Alain Verloes, Gaelle Vieville, Catherine Yardin, François Vialard, Martine Doco‐Fenzy

**Affiliations:** ^1^ Service de Génétique CRMR AnDDI‐Rares, CHU Reims Reims France; ^2^ Centre de Référence des Malformations et Maladies Congénitales du Cervelet, Département de Génétique et Embryologie Médicale APHP, Hôpital Trousseau Paris France; ^3^ Cardiologie pédiatrique et congénitale, CHU Reims Reims France; ^4^ Service de pédiatrie Pôle Femme Parents Enfants, CHU Reims Reims France; ^5^ CReSTIC/EA 3804 URCA Reims France; ^6^ University of Brest Inserm, EFS, UMR 1078, GGB Brest France; ^7^ Département de Génétique Hôpital Robert Debré Paris France; ^8^ Service de Génétique Hospices Civils de Lyon Bron France; ^9^ Laboratoire de Cytogénétique, CHU Dijon Dijon France; ^10^ AP‐HP Sorbonne Université Département de Génétique Médicale, Hôpital Armand Trousseau Paris France; ^11^ Département de Génétique Médicale, Maladies Rares et Médecine Personnalisée, Génétique clinique, CHU Montpellier Université Montpellier, Centre de référence anomalies du développement SOOR Montpellier France; ^12^ Département de Génétique et Procréation Hôpital Couple Enfant, CHU Grenoble‐Alpes Grenoble France; ^13^ Genetic Epigenetic and Therapies of Infertility team Institute for Advanced Biosciences, Inserm U 1209, CNRS UMR 5309, Université Grenoble Alpes Grenoble France; ^14^ Centre de Génétique Chromosomique GH de l'Institut Catholique de Lille‐Hopital Saint Vincent de Paul Lille France; ^15^ Laboratoire de Cytogénétique Constitutionnelle APHP. Centre‐Université Paris Cité site Cochin Paris France; ^16^ Plateforme ChromoStem, Unité de génétique chromosomique, Département de génétique moléculaire et cytogénomique, CHU de Montpellier Université de Montpellier Montpellier France; ^17^ Service de Génétique, CHU Caen Université Caen Normandie Caen France; ^18^ Département de Génétique Centre Hospitalier Intercommunal Poissy‐St‐Germain‐en‐Laye Poissy France; ^19^ Département de Génétique; Centre de Référence Déficience Intellectuelle de Causes Rares APHP Sorbonne Université, GH Pitié‐Salpêtrière Paris France; ^20^ Service de Génétique Médicale, CHU de Nantes Nantes France; ^21^ National Center of Genetics Laboratoire National de Santé Dudelange Luxembourg; ^22^ Oncobiologie Génétique Bioinformatique, CHU de Besançon Besançon France; ^23^ Service de neuropédiatrie, Hôpital Armand Trousseau Groupe Hospitalier Universitaire de l'Est Parisien Paris France; ^24^ Service de génétique médicale, CH du Mans Le Mans France; ^25^ Laboratoire de Génétique Chromosomique, Département de Génétique Médicale AP‐ HM Marseille France; ^26^ Service de Cytogénétique Médicale CHU de Clermont‐Ferrand Clermont‐Ferrand France; ^27^ Service de Génétique Médicale et Biologie de la Reproduction, CHU de Brest Brest France; ^28^ Department of Cytogenetics and clinical genetics, Limoges University Hospital University of Limoges Limoges France; ^29^ RHuMA, UMR BREED INRAE‐UVSQ‐ENVA Montigny‐le‐bretonneux France; ^30^ Service de génétique médicale, CHU de Nantes Nantes France; ^31^ L'institut du thorax INSERM, CNRS, UNIV Nantes, CHU de Nantes Nantes France

**Keywords:** 1p36 deletion syndrome, chromosomal deletion, genotype–phenotype correlation, monosomy 1p36

## Abstract

Chromosome 1p36 deletion syndrome (1p36DS) is one of the most common terminal deletion syndromes (incidence between 1/5000 and 1/10,000 live births in the American population), due to a heterozygous deletion of part of the short arm of chromosome 1. The 1p36DS is characterized by typical craniofacial features, developmental delay/intellectual disability, hypotonia, epilepsy, cardiomyopathy/congenital heart defect, brain abnormalities, hearing loss, eyes/vision problem, and short stature. The aim of our study was to (1) evaluate the incidence of the 1p36DS in the French population compared to 22q11.2 deletion syndrome and trisomy 21; (2) review the postnatal phenotype related to microarray data, compared to previously publish prenatal data. Thanks to a collaboration with the ACLF (Association des Cytogénéticiens de Langue Française), we have collected data of 86 patients constituting, to the best of our knowledge, the second‐largest cohort of 1p36DS patients in the literature. We estimated an average of at least 10 cases per year in France. 1p36DS seems to be much less frequent than 22q11.2 deletion syndrome and trisomy 21. Patients presented mainly dysmorphism, microcephaly, developmental delay/intellectual disability, hypotonia, epilepsy, brain malformations, behavioral disorders, cardiomyopathy, or cardiovascular malformations and, pre and/or postnatal growth retardation. Cardiac abnormalities, brain malformations, and epilepsy were more frequent in distal deletions, whereas microcephaly was more common in proximal deletions. Mapping and genotype–phenotype correlation allowed us to identify four critical regions responsible for intellectual disability. This study highlights some phenotypic variability, according to the deletion position, and helps to refine the phenotype of 1p36DS, allowing improved management and follow‐up of patients.

## 1 INTRODUCTION

Chromosome 1p36 deletion syndrome (1p36DS) (MIM:607862 and ORPHA:1606) is one of the most common terminal deletion syndromes with an incidence classically reported as ranging from 1/5000 and 1/10,000 live births (Heilstedt, Ballif, Howard, Kashork, & Shaffer, 2003; Shapira et al., 1997; Shimada et al., 2015). It represents about 0.5–1.2% of cases with syndromic intellectual disability (ID) (Battaglia, 1993; Guterman et al., 2019; Heilstedt, Ballif, Howard, Lewis, et al., 2003).

This syndrome is due to a heterozygous deletion of part of the short arm of chromosome 1. Pure terminal deletions (52–67%), interstitial deletions (10–29%), derivative chromosomes (7–16%), and more complex rearrangements (12%) have been reported (Battaglia, 1993; Rocha et al., 2016). Chromosome 1p36DS can be diagnosed by conventional cytogenetic analysis, fluorescence in situ hybridization (FISH) analysis, and chromosomal microarray analysis (CMA). CMA has been, until recently, the most suitable method (Battaglia, 1993; Cunha et al., 2014; Rocha et al., 2016), and whole exome or genome sequencing with copy number variant analysis may now also be available for identifying 1p36 deletion (Toshimitsu et al., 2019).

Although the presence of repetitive DNA sequences (Rocha et al., 2016) in the 1p36 region has been proposed to explain the deletion occurrence, the underlying mechanisms remain elusive. In 2003, Heilstedt and colleagues have demonstrated the presence of numerous different breakpoint clusters resulting in the deletion of variable size fragments (Heilstedt, Ballif, Howard, Lewis, et al., 2003).

This syndrome is most commonly characterized by typical craniofacial features such as straight eyebrows; deep‐set eyes; midface hypoplasia; wide and flat nasal bridge; large, late‐closing anterior fontanel; microbrachycephaly and posteriorly rotated, low‐set, and asymmetric ears (Battaglia, 1993; Rocha et al., 2016). Other major clinical features are: developmental delay/ID (100%), hypotonia (95%), epilepsy (44–70%), congenital heart defect (43–71%), cardiomyopathy (22–27%), brain abnormalities (88%), hearing loss (47–82%), eyes/vision problem (52%), and short stature (Battaglia, 1993; Jordan et al., 2015). Females are more affected than males (Battaglia, 1993; Heilstedt, Ballif, Howard, Lewis, et al., 2003; Shimada et al., 2015).

The deletions size is variable (up to 16.5 Mb) and not always associated with phenotype severity (Rocha et al., 2016). Indeed, the correlation between deletion size and the number of observed clinical features has been debated (Gajecka et al., 2007). It is a contiguous gene deletion syndrome with multiple congenital anomalies and ID (Shapira et al., 1997), probably caused by haploinsufficiency of several genes (Heilstedt, Ballif, Howard, Lewis, et al., 2003). Distal and proximal 1p36 critical regions have been described in the literature. They encompass some of these genes whose haploinsufficiency may be responsible for the phenotype, especially: *GABRD, KCNAB2, SKI, RERE, UBE4B, GNB1, PRDM16* (Jordan et al., 2015; Petrovski et al., 2016, p. 1). Other genes may contribute to the phenotype such as *SPEN* for which haploinsufficiency has been recently associated with neurodevelopmental disorders (Radio et al., 2021).

Approximately 600 patients have been described in the literature (excluding DECIPHER). Through several published European, American, and Japanese cohorts (Bahi‐Buisson et al., 2008; Battaglia et al., 2008; Heilstedt, Ballif, Howard, Lewis, et al., 2003; Shimada et al., 2015) the 1p36DS has been characterized but due to clinical and genetic heterogeneities (variability of size and position of deleted segment, variability of clinical features, existence of mosaicism), establishing a genotype–phenotype correlation in 1p36 deletion remains a challenge.

Thanks to a collaboration with the Association des Cytogénéticiens de Langue Française (ACLF, the French‐Speaking Cytogeneticists Association: www.eaclf.org), we have collected data of 86 patients postnatally diagnosed with 1p36DS. Data from the corresponding prenatal observations have been previously published (Guterman et al., 2019). To the best of our knowledge, we reported here the second‐largest cohort of 1p36DS patients in the literature, performing mapping, and genotype–phenotype correlation.

The aim of the present study was (1) try to assess all cases of 1p36 deletion diagnosed in France and with previously reported prenatal cases, try to evaluate its incidence; (2) review the phenotype, related to CMA data and help to establish a link between genotypes and phenotypes associated with this genetic syndrome to provide more information to families.

## 2 PATIENTS AND METHODS

### 2.1 Study design and population

A multicenter, nationwide, retrospective study was set up to collect data on patients with 1p36DS with the cooperation of the ACLF.

Ninety‐one patients diagnosed postnatally between 1996 and 2018 using various diagnostic methods (conventional karyotyping, FISH analysis, Multiplex Ligation‐dependent Probe Amplification (MLPA), or CMA) were reported by 18 French laboratories out of the 37 surveyed centers. The following data were requested by a questionnaire addressed to the referring geneticist: indication for genetic testing, clinical information, age at diagnosis, technique used for diagnosis, and mode of inheritance.

Five patients were excluded from the clinical analysis: two patients for whom genetic and clinical information was lacking, one patient with a derivative chromosome one resulting from an unbalanced translocation (with 1p36 deletion associated with a 6 Mb chromosome 16 duplication), two other patients with very small deletions (41 and 166 kb) encompassing the *GNB1* gene.

### 2.2 Mapping

Mapping data were obtained from either BAC‐arrays, SNP‐arrays, or array‐CGH (aCGH) for most of them. A map of CMA results was generated using the University of California Santa Cruz (UCSC: https://genome.ucsc.edu/) genetic tools, UCSC Genome Browser (build GRCh37/hg19), with the creation of two groups according to deletion position. Distal and proximal 1p36 critical regions (respectively chr1:1‐6,289,973 and chr1:8,395,179‐11,362,893) were described in the literature (Jordan et al., 2015). The middle of these critical regions was used to create the two groups: Group A with distal deletions and Group B with proximal deletions. Deletions were considered apparently terminal when their boundary corresponded to the first or second probe of the array used and/or if a telomeric probe was deleted by FISH. When these data were not available, deletions were considered interstitial if they started above 852,863 kb. 1p36 deletion associated with other deletion or duplication on chromosome 1 were considered as complex rearrangements.

### 2.3 Estimated incidence

To estimate the number of cases diagnosed per year between 2012 and 2016, the prenatally diagnosed 1p36DS cases, previously published in the same geographic origin population by Guterman and colleagues, were used (Guterman et al., 2019). Since not all centers invited to collaborate participated, we focused on our region in France to estimate the incidence of 1p36DS assuming that the cases were exhaustively analyzed. We used the official data of live births per year in this region (Insee data: National Institute of statistics and national studies).

### 2.4 Statistical analysis and clinical data

Clinical data were described using mean and median for quantitative variables and number and percentage for qualitative variables. Clinical data were studied between the two groups using univariate analyses (Fisher's exact tests). Statistical analyses were performed with Epi Info™ version 3.5.4 (CDC). For all tests, *p* < 0.05 were considered to be statistically significant.

Patients' photographs were analyzed using Face2gene (FDNA, Inc.: https://www.face2gene.com/) to evaluate facial features.

## 3 RESULTS

### 3.1 Study population

*Gender*: Out of 86 patients postnatally diagnosed in our study, gender was known in 80 patients, with 46 females and 34 males (sex ratio = 0.74).

*Age at diagnosis*: The patient's age at diagnosis was known in 85 cases. The mean and median age ± SD (range) were respectively 6.12 and 2 ± 8.3 (0.005–33.7) years old (from 2 days to 33 years). Among all cases, 9 patients were newborns (age below 28 days), 34 infants (age between 1 month and 2 years), 15 young children (age between 2 and 6 years), 12 older children (age between 7 and 12 years), 8 adolescents (age between 13 and 19 years), and 7 adults (age > 20 years).

*Mortality*: Information about survival was known in 51 cases. Among these 51 patients, 8 children were dead (15.7%), and 6 of them died before 2 years old. Deletions sizes of these patients (del40, del56, del43, del76, del21, del25, del37, and del86) varied from about 3.7 to 8.3 Mb (Table S1). The cause of death was known in three cases with cardiogenic shock in a patient with dilated cardiomyopathy (del40) and cardiac decompensation in a patient with cardiomyopathy (del25). All these patients (8/8) suffered from heart defects: dilated cardiomyopathy (3), left ventricular non‐compaction (1), ventricular septal defect (3), atrial septal defect (1), tetralogy of Fallot (2), patent ductus arteriosus (2), overriding aorta (1), and abdominal aortic hypoplasia (1). Furthermore, 6/7 (85.7%) presented cerebral abnormalities (left hemispheric atrophy, pachygyria, cortical dysplasia, ballooned ventricles, white matter anomalies, square aspect of the frontal and occipital horns, increased bifrontal pericerebral spaces, anomalies of the cerebellum tent, ventriculomegaly, and cerebral cysts), and 5/6 (83%) presented epilepsy (epileptic encephalopathy, West syndrome, tonic–clonic seizures, and cheiro‐oral seizures).

### 3.2 Diagnostic methods and molecular cytogenetics findings

1p36DS diagnosis was made by CMA in 72 patients, FISH alone in 11 patients, MLPA in 1 patient, MLPA associated with FISH in 1 patient, and minisatellite probes associated with FISH in 1 patient. We found so‐called terminal deletions (55), interstitial deletions (24), and derivative chromosomes (1). In five patients, CMA allowed to bring out complex rearrangements (1p36 deletion associated with other deletion or duplication on chromosome 1). In our cohort, minimum and maximum deletions sizes varied from 532 kb to 11.8 Mb (Table S1).

**FIGURE 1:**
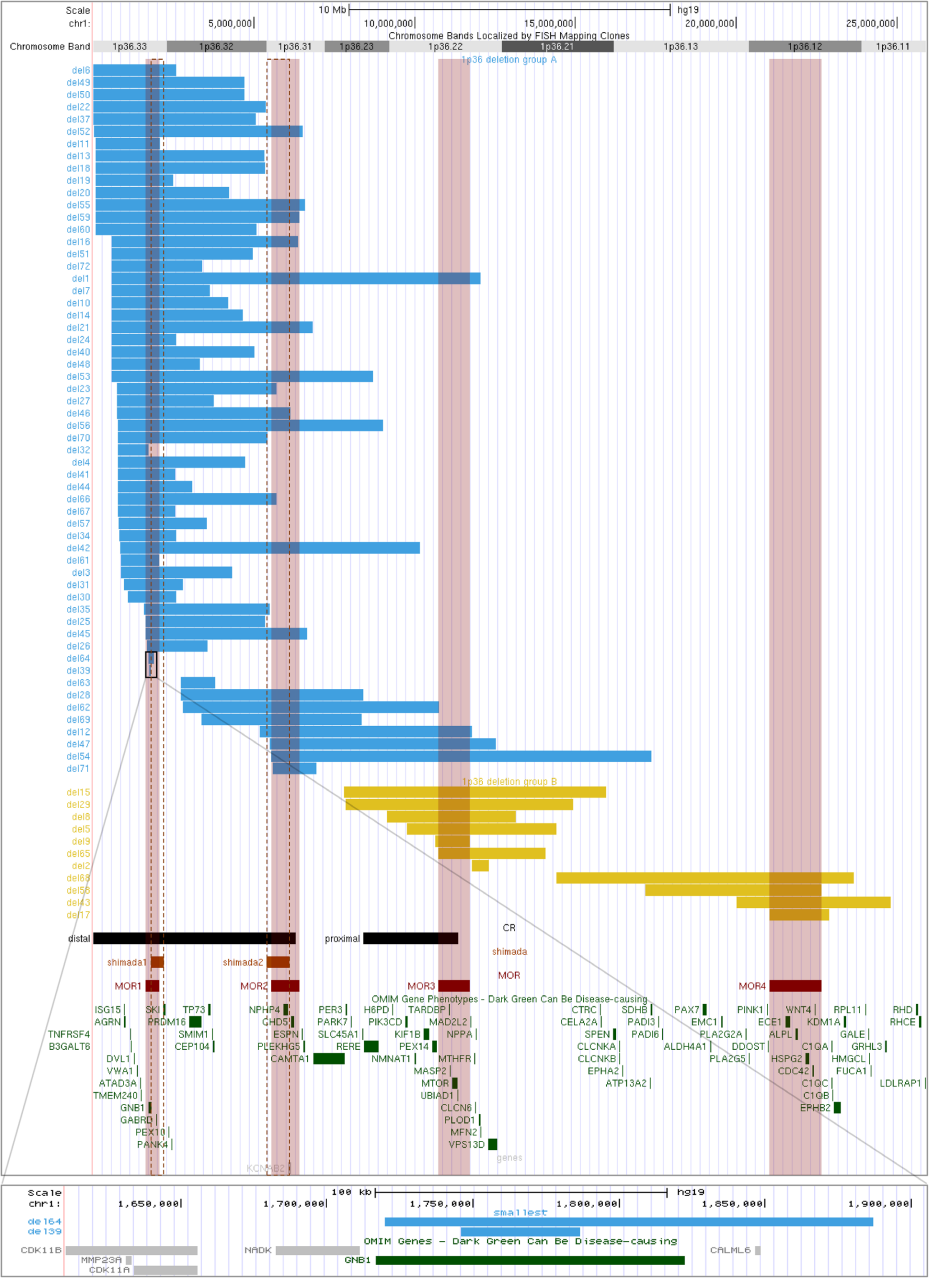
Mapping of deletions diagnosed by CMA using UCSC genome browser (build GRCh37/hg19) and *OMIM* genes included in this region. Blue bars represent Group A deletions (*n* = 56 patients), yellow bars Group B deletions (*n* = 11 patients), black bars distal and proximal critical regions described in the literature, orange bars and orange vertical dotted lines critical regions described by Shimada et al., red bars and red translucent vertical lines represent the proposed four MORs: MOR1: 1,619,654‐2,057,167; MOR2: 5,528,518‐6,414,084; MOR3: 10,732,711‐11,718,611; MOR4: 21,035,150‐22,652,664. The lower box shows a zoom on the two smallest deletions excluded from the cohort and the *GNB1* gene. CMA, chromosomal microarray analysis; MOR, minimal overlapping region

Out of the 86 patients included in the study, CMA was performed on 72 patients. Among them, additional aberrations were reported in six patients and summarized in Table S1. CMA data allowed us to align 67 deletions and to generate a map (Figure 1) using the UCSC Genome Browser (build GRCh37/hg19). Mapping allows us to visualize several groups of deletions limited by breakpoints, each of them facing segmental duplications (Figure S1).

The cohort was divided into two groups according to the deletion position: Group A including 56 patients, and Group B including 11 patients (Figure 1). We delineated four regions proposed as responsible for ID (illustrated in Figures 1 and S2) corresponding to minimal overlapping regions (MOR) common to the largest number of deletions. The first one covered approximately the 1.6–2 Mb region, the second the 5.5–6.4 Mb region, the third the 11–11.7 Mb region, and the last, the 21–22.7 Mb region (Figure 1).

### 3.3 Inheritance

Of the 54 patients for whom a parental analysis was performed: the 1p36 deletion was found de novo in 50 patients, inherited in two cases (one case maternally inherited and one case paternally inherited), resulted from an unbalanced translocation in one case (plus another case excluded from the study), and one 1p36 deletion was found in mosaic (20–50%, tissular).

### 3.4 Clinical findings

**FIGURE 2:**
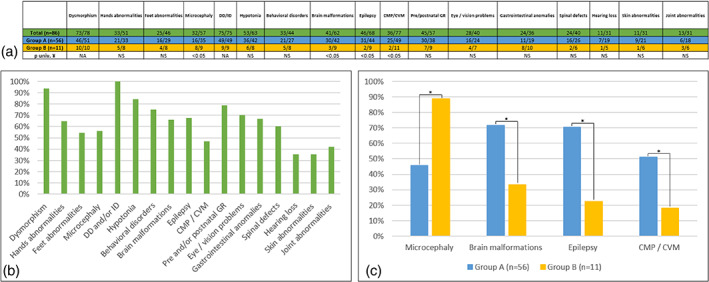
Table of absolute values (a) and graph summarizing the proportions in percent (b, c) of the main clinical signs in all patients (green), Group A (blue), and Group B (yellow). CMP, cardiomyopathy; CVM, cardiovascular malformations; DD, developmental delay; GR, Growth retardation; ID, Intellectual disability; NA, not appropriate; NS, not significant; univ, Univariate, ¥: Fisher's exact tests. * *p* < .05

Clinical features in all patients, in Group A (distal deletions), and Group B (proximal deletions) were summarized in Figure 2. Developmental delay or ID (100%, 75/75), facial dysmorphism (94%, 73/78), hypotonia (84%, 53/63), epilepsy (68%, 46/68), brain malformations (66%, 41/62), and cardiomyopathy or cardiovascular malformations (47%, 36/77) were major features in our cohort.

We also found: pre‐ and/or post‐natal growth retardation in 45 patients (45/57), behavioral disorders in 33 patients (33/44), microcephaly in 32 patients (32/57), eye/vision problems in 28 patients (28/40), hands (33/51), and feet (25/46) abnormalities, gastrointestinal anomalies in 24 patients (24/36) and also spinal defects (24/40).

#### 3.4.1 Developmental delay or intellectual disability

Data were available in 75 patients, and developmental delay or ID was reported in 100% of patients (75/75). The severity degree of this delay was unknown in most cases. Walking (data was available in 17 patients) was acquired for the earliest at 16 months and was not acquired at the time of the consultation for three patients at 3, 5, and 10 years, respectively. A regression of acquisitions was reported in four patients (details in Table S3). Language delay was also a frequent feature (according to the available data, two patients have no language at 17 years and 5 years, respectively). One patient was described with mild ID (del61) and was autonomous in daily life.

Thirty‐three patients presented behavioral disorders (data was available in 44 patients), including aggressivity, self‐injury/self‐aggressivity, temper tantrums, low frustration tolerance, social interaction difficulties, and stereotypies.

#### 3.4.2 Dysmorphism

**FIGURE 3:**
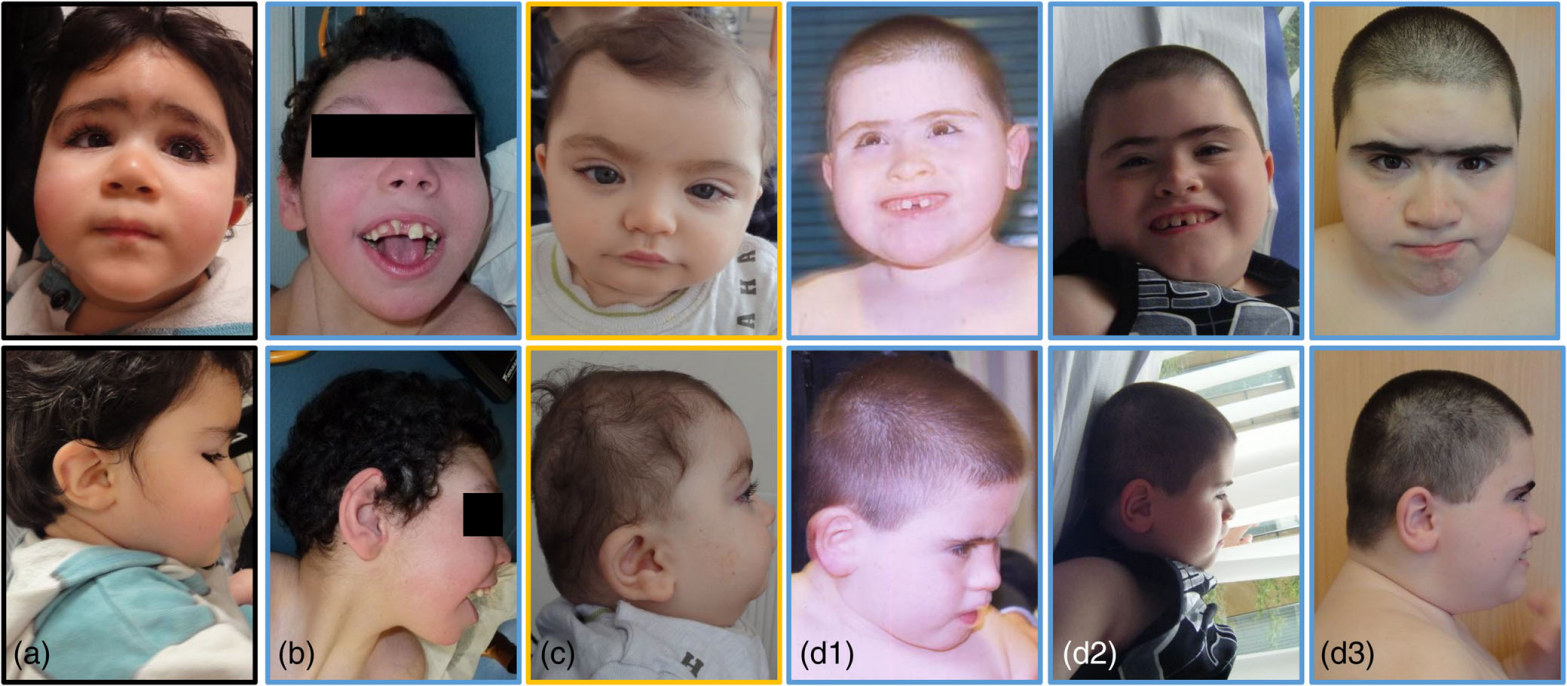
Four patients with 1p36 deletions. Blue and yellow frames corresponding to Groups A and B, respectively. Patient a (2 years and a half) corresponding to del85, patient b to del62, patient c (1 year) to del2, and patient d to del50: 8 years (d1), 10 years (d2), 15 years (d3)

Data were available in 78 patients with details in 69 patients. Facial dysmorphism was reported in 94% (73/78) (Figure 3) with: mostly horizontal and straight eyebrows (46%, 32/69), enophtalmia (39%, 27/69), blepharophimosis (13%, 9/69), epicanthal folds (12%, 8/69), low‐set (27%, 19/69), and posteriorly rotated (13%, 9/69) ears, sometimes also sticky‐out ears (12%, 8/69), midface hypoplasia (16%, 11/69), short nose (12%, 8/69), microstomia (23%, 16/69) with thin lips (17%, 12/69), and brachycephaly (13%, 9/69). Nineteen patients' photographs were analyzed using Face2gene (FDNA, Inc.), without clinical information. This tool proposed 1p36DS as the most likely diagnosis in 10 patients and in the top 10 and 30 diagnoses in four patients and one patient, respectively. 1p36DS was not proposed at all as a diagnosis in four patients.

#### 3.4.3 Brain malformations

Data were available in 62 patients. About 66% (41/62) of patients presented brain malformations, including corpus callosum abnormalities for 13 patients (13/62) and ventricular dilation for 12 patients (12/62). We also observed polymicrogyria, pachygyria, cortical dysplasia, heterotopia, cerebral atrophy, delay in myelination, and white matter anomalies (Tables S2 and S4). Deletions were mapped in 33 patients.

#### 3.4.4 Epilepsy

(Data were available in 68 patients) was found in 46 patients (deletions were mapped in 33 patients) and associated with brain malformations in 28 patients. Interestingly brain malformations were more frequent in Group A (30/42, Group A; 3/9, Group B) as well as epilepsy (31/44, Group A; 2/9, Group B) (*p* < 0.05).

#### 3.4.5 Cardiomyopathy or cardiovascular malformations

Data were available in 77 patients. Cardiovascular malformations or cardiomyopathy were reported in 47% (36/77). Of these 36 patients, deletions were mapped in 27 of them. Structural heart defects were observed in 32% of patients with mostly ventricular septal defect (VSD: 11/77, 14%) and patent ductus arteriosus (PDA: 9/77, 12%). Cardiomyopathy (dilated cardiomyopathy, hypertrophic cardiomyopathy, left ventricular non‐compaction, and/or left ventricular dysfunction) were noted in 21% of patients (left ventricular non‐compaction and dilated cardiomyopathy in 7/77 patients (9%) and 6/77 patients (8%), respectively). Four patients presented both structural heart defects and cardiomyopathy. Cardiac abnormalities were more frequent in Group A (25/49) than in Group B (2/11) (*p* < 0.05). Cardiac abnormalities were found in 77.8% of newborns (7/9), 59.4% of infants (19/32), 33.3% of young children (5/15), 37.5% of older children (3/8), 14.3% of adolescents (1/7), and 20% of adults (1/5).

#### 3.4.6 Hands and feet abnormalities

(Data were available in 51 and 46 patients): short hands, bilateral talus valgus, clinodactyly, flat feet, brachydactyly, and transverse palmar crease were reported.

#### 3.4.7 Microcephaly

Data were available in 57 patients. Microcephaly was found in 32 patients and was more frequent in Group B (8/9) than in Group A (16/35) (*p* < 0.05). Deletions were mapped in 24 patients. It was associated with brain abnormalities in 16 patients.

#### 3.4.8 Other abnormalities reported

Hearing loss (11), skin abnormalities (11), joint abnormalities (13), limbs abnormalities (9), overweight (9), lungs abnormalities (8), abnormalities of the external genitalia (8), hypertrichosis (7), renal defects (6), early puberty (5), hypertonia/ pyramidal syndrome (5), hypothyroidism (4), hepatic defects (3), and hematologic disorders (2). All clinical signs reported, are shown in Tables S2 and S4.

### 3.5 Severity

In our cohort, deletions over 10 Mb were associated with a more severe presentation. Two patients had deletions larger than 10 Mb. The first one presented dysmorphism; left ventricular non‐compaction; ventricular septal defect; microcephaly; large anterior fontanel; corpus callosum hypoplasia; delayed myelination; hypotonia, feet abnormalities; growth retardation; hearing loss, and psychomotor delay. The second carried the largest deletion: 11.8 Mb (del54). Interestingly, this patient had neither cardiomyopathy nor congenital heart defect, microcephaly, epilepsy, or growth delay but he presented intra‐uterine growth delay. It was an interstitial deletion that did not include the *GABRD*, *SKI*, or *PRDM16* genes. Nevertheless, he exhibited facial dysmorphism, partial agenesis of the corpus callosum, hypotonia, spinal abnormalities, short fingers, hypertrichosis, feeding difficulty with gastroesophageal reflux requiring a gastrostomy, myopia, hearing loss, and sleep apnea syndrome requiring a tracheotomy. He presented psychomotor delay (did not walk at 10) and mild to severe ID. So, even if he did not present all the major features of 1p36 deletion, he had a severe neurodevelopmental phenotype.

### 3.6 Number of cases diagnosed by year

Using previously published data, we calculated the number of new cases of 1p36DS per year between 2012 and 2016. As shown in Table 2, an average of, at least, 10 cases per year was reported. In our region, four cases of 1p36DS were diagnosed between 2012 and 2016, and an average of 14,930 live births per year was estimated, that is, approximately 1 case per 18,000 live births.

## 4 DISCUSSION

With the cooperation of the members of the ACLF, we reported 86 patients with 1p36DS diagnosed postnatally between 1996 and 2018. According to the deletion position, we have delineated two groups: 56 patients with distal deletions (Group A) and 11 patients with more proximal deletions (Group B). Patients in our cohort presented mainly: facial dysmorphism, microcephaly, developmental delay or ID, hypotonia, epilepsy, brain malformations, behavioral disorders, cardiomyopathy or cardiovascular malformations, pre‐ and/or postnatal growth retardation. Cardiac abnormalities, brain malformations, and epilepsy appeared to be more frequent in Group A than in Group B, whereas microcephaly seemed to be more common in Group B.

### 4.1 Limitation of the study

This large retrospective study has the inconvenience of lacking some clinical data, however, it gives information about the frequency of the deletion 1p36, in continuum with our previous del1p36 prenatal study, in the same population.

### 4.2 Estimated incidence

We estimated an average of 10 cases of 1p36DS diagnosed per year in France. However, only 18 laboratories reported cases, so we probably could not assess all the cases of 1p36DS in France; thus, the number of cases per year is probably underestimated in our cohort. However, our results give an approximation of the incidence of this syndrome, about 1 case per 18,000 live births, which seems to be slightly lower than that described in the literature (between 1/5000 to 1/10,000 live births) (Heilstedt, Ballif, Howard, Lewis, et al., 2003; Shapira et al., 1997; Shimada et al., 2015). Poirsier and colleagues with the cooperation of the ACLF performed the same study about 22q11.2 deletion syndrome and reported an average of 108 new cases per year compared to 2758 trisomy 21 cases. Thus, we can compare the incidence of 1p36 deletion, 22q11.2 deletion syndrome, and trisomy 21 in the French population (Table 2) and even if the study period is different, the 1p36DS seems to be much 10 less frequent than 22q11q.2 deletion and less frequent than Down syndrome (Poirsier et al., 2016).

### 4.3 Diagnostic methods and molecular cytogenetics findings

The majority of patients analyzed in this study were females (47 females and 36 males), as already described in the literature (Battaglia, 1993; Heilstedt, Ballif, Howard, Lewis, et al., 2003; Shimada et al., 2015). In our cohort, the 1p36 deletion was de novo in the majority of patients consistent with the literature, however evidence of germline mosaicism has also been described (Gajecka et al., 2010; Nistico' et al., 2020). In some cases, the diagnosis may have been easily made by targeted analyses in patients with typical signs of the 1p36 deletion. About 75% of the patients in our cohort were diagnosed, mostly using aCGH, before 7 years old, and the median age was about 2 years old. Patients less than 2 years of age had clinical features similar to those presented by all patients. However, in patients who were diagnosed later, cardiac abnormalities were less frequent.

According to the deletion mapping and critical regions described in the literature, we divided the cohort into two groups: 56 patients with distal deletions (Group A) and 11 patients with more proximal deletions (Group B), so classical distal deletion is more frequent in our cohort (Figure 1). The underlying mechanisms explaining the occurrence of 1p36DS remain elusive but the presence of repetitive DNA sequences in the 1p36 region has been proposed as an explanation (Rocha et al., 2016). We observed that breakpoints were located opposite segmental duplications (Figure S1). We can hypothesize that misalignment of these segmental duplications during non‐allelic homologous recombination may lead to 1p36 microdeletion.

### 4.4 Phenotype severity

In our study, all patients who died suffered from heart defects, and among them, two died from cardiogenic shock, and heart failure. Shimada and colleagues have reported two deceased patients, one of whom died at 10 months old of heart failure (Shimada et al., 2015). So 1p36DS may be a life‐threatening condition and it appears that the vital prognosis is related to congenital heart defect or functional cardiac abnormalities.

No correlation between deletion size and the number of observed clinical features has been previously described (Gajecka et al., 2007). However, in our cohort, deletions over 10 Mb were associated with a severe presentation, especially a severe neurodevelopmental phenotype. We described one patient exhibiting mosaicism (del22), interestingly, this patient has a severe presentation with brain abnormalities, epileptic encephalopathy, and ID. Shimada and colleagues also described three patients with mosaic 1p36 deletion with moderate and severe ID (Shimada et al., 2015).

### 4.5 Developmental delay

Developmental delay is a constant feature of the 1p36DS, in variable degree (Battaglia, 1993). Indeed, in the present study, one patient was described with mild ID (del61) and autonomous in daily life while some patients had never acquired the ability to walk. Several genes in 1p36 region may contribute to the ID and psychomotor delay as previously reported (Battaglia, 1993; Jordan et al., 2015; Radio et al., 2021; Shimada et al., 2015). Attempting to compare our results with 1p36 deletion literature, we superimposed the deletions of the patients and we identified four MORs from 1pter to 1cen (Figures 1 and S2). We remarked that the location of MOR1 (1.6–2 Mb region), and MOR2 (5.5–6.2 Mb) matched with the two proposed responsible regions for ID reported by Shimada et al. (2015) namely 1.8–2.2 Mb encompassing *GNB1* (Figure 1) and 5.4–6.1 Mb (encompassing *KCNAB2* and *CHD5*). In Shimada's report, most patients had the first SRO and some had the first and second SRO, authors explained that severe neurodevelopmental prognosis may be provided by haploinsufficiencies of *KCNAB2* and *CHD5*. Interestingly in our cohort, all patients presented ID, and some have deletions of MOR2 (*KCNAB2* and *CHD5*) not encompassing MOR1 (*GNB1*). This suggests that genes in MOR2 may also contribute to the neurological phenotype observed in the 1p36DS. Petrovski et al. have shown that de novo mutations in *GNB1* cause severe neurodevelopmental disability, hypotonia, and seizures. Two unique patients (del64 and del39) carried very small deletions (41 and 166 kb) encompassing only the *GNB1* gene (Figure 1). They shared some characteristics with patients described in Petrovski's publication: global developmental delay (and ID), hypotonia, and language delay (Petrovski et al., 2016). Interestingly, the deletion of patient 32, encompassing partly *GNB1*, is reported with dysmorphism, seizures, hypotonia, myelination delay, ventriculomegaly, and developmental delay without any cardiac malformations. Furthermore, heterozygous variants in *CHD5* have recently been associated with developmental delay, ID, behavioral disorder, and epilepsy (Parenti et al., 2021, p. 5) reinforcing the hypothesis that *CHD5* may play a role in ID in the 1p36DS.

**FIGURE 4:**
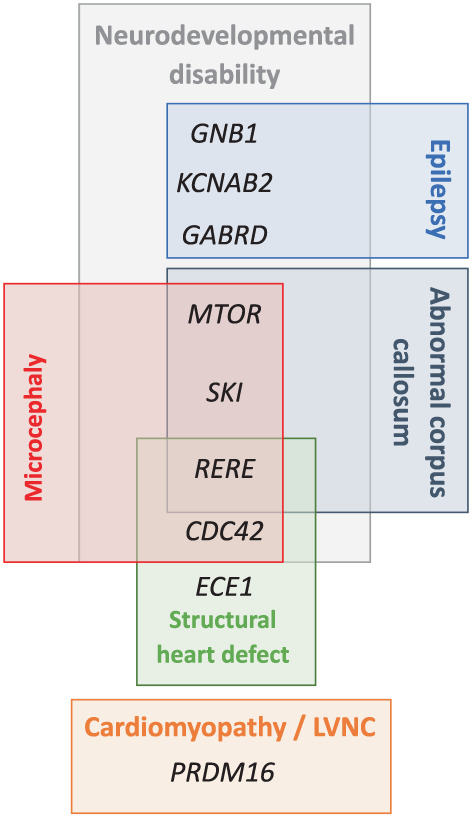
Major candidate genes that may explain the 1p36 deletion syndrome's phenotype in our cohort

Besides *GNB1* and *CHD5*, several genes may contribute to the ID and psychomotor delay, such as *GABRD* (MIM:137163), *SKI* (MIM:164780), *KCNAB2* (MIM:601142), *RERE* (MIM:605226), *UBE4B* (MIM:613565), *SPEN* (MIM:613484), and *CDC42* (MIM:116952) (Figures 1 and 4) (Jordan et al., 2015; Radio et al., 2021). Kang et al. (2007) identified a proximal critical region (PCR) thanks to the description of five patients (8.4–11.3 Mb) among them a patient with a 2.97 Mb deletion. We delineated a smaller MOR (MOR3) in our cohort (11–11.7 Mb region) which could even be reduced to 11–11.4 Mb region using the boundaries of the first PCR described by Kang et al. (2007) (Figure 1). This region was proposed as responsible for ID, and it contains the *MTOR gene (MIM:601231)*. Finally, we report another even more proximal MOR (MOR4) (21–22.7 Mb region) including the *CDC42* gene. Heterozygous variants of *CDC42* have been reported in ID (with specific dysmorphic facial features and notably microcephaly and heart defects) (Takenouchi et al., 2016). The gene *SPEN (chr1:15.8–15.9(hg18), 16.1–16.2(hg19)*) recently published as causing neurodevelopmental disorder (Radio et al., 2021) was deleted in only two patients in our cohort (del68 and del54) both sharing deletion of long segments (including MOR3 or MOR4, respectively). The *RERE and UBE4B* not included in any of our four MORs were still deleted in nine patients and present in the PCR described by Kang et al. (2007). Thus, the neurodevelopmental delay and the intellectual deficiency are probably the product of different genes and modulating 1p36 regions.

### 4.6 Epilepsy

Epilepsy was described in 44% (Battaglia et al., 2008) to 70% (Shimada et al., 2015) of patients in the literature and it was also a frequent neurological feature in our study (Table 1). We found more patients with epilepsy in Group A than in Group B. Indeed, *GABRD* and *KCNAB2* are located within the distal critical region and MOR1 and 2 (Figure 1). Gamma‐aminobutyric acid receptor delta (*GABRD*) encodes for ligand‐gated chloride channels for GABA (gamma‐aminobutyric acid), the major inhibitory neurotransmitter in the mammalian brain (Windpassinger et al., 2002). In 2018, Bhat and colleagues found that polymorphism in the *GABRD* gene, as well as other GABAA receptor genes, may be associated with juvenile myoclonic epilepsy and Lennox–Gastaut syndrome (Bhat et al., 2018). The *KCNAB2* (potassium voltage‐gated channel subfamily A regulatory beta subunit 2) gene has been associated with epilepsy in 1p36DS (Heilstedt et al., 2001), and a recent study also supports this hypothesis (Shimada et al., 2015). We found 28 epileptic patients whose deletions included *GABRD* among 47 and 8 epileptic patients whose deletions encompassed *KCNAB2* among 18, also that may suggest a reduced penetrance of these genes or the intervention of other factors as stochastic effects. The haploinsufficiency of more than one gene could promote the onset of epilepsy. Five out of 10 patients with deletions including both *GABRD* and *KCNAB2* had epilepsy in our cohort.

**TABLE 1:** Cardinal clinical characteristics of 1p36DS

Cardinal clinical characteristics	Heilstedt, Ballif, Howard, Lewis, et al. (2003) (*n* = 61)	Battaglia et al. (2008) (*n* = 60)	Shimada et al. (2015) (*n* = 50)	Present study (*n* = 86)
Dysmorphism	100%	100%	Most of patients	94%
DD or ID	100%	100%	98%	100%
Hypotonia	87%	95%	92%	84%
Epilepsy	48%	44%^a^	70%	68%
Cardiac abnormalities Congenital heart defects Cardiomyopathy	43% 23%	71% 27%	69% 22%	32% 23%
Brain abnormalities	NA	88%	NA	66%
Hearing loss	82%	47%	39%	35%
Eyes/vision problem	83%	52%	NA	70%
Short stature	NA	96%^b^	NA	79%^c^

Abbreviations: DD, developmental delay; ID, intellectual disability; NA, not available.

^a^ Epilepsy was observed in 58,2% in the 91 patients reported by Bahi‐Buisson et al.

^b^ Prenatal growth retardation.

^c^ Pre‐ and/or postnatal growth retardation.

**TABLE 2:** Number of cases of 1p36 deletion syndrome diagnosed per year pre‐ and postnatally in the collaborating laboratories, compared with the number of cases of 22q11.2 deletion syndrome and 21 trisomy from previous study

	**2012**	**2013**	**2014**	**2015**	**2016**	**Average per year**
1p36 DS						
Postnatally diagnosis	8	8	9	10	7	8.4
Prenatally diagnosis^a^	1	0	4	1	2	1.6
Total	9	8	13	11	9	10
	**2010**	**2011**	**2012**			**Average per year**
22q11DS^b^						
Postnatally diagnosis	74	65	78			108
Prenatally diagnosis	31	37	41			
	**2010**	**2011**	**2012**			**Average per year**
Trisomy 21^b^						
Postnatally diagnosis	435	535	488			2758
Prenatally diagnosis	2369	2477	1971			

^a^ Data from Guterman's study.

^b^ Data from Poirsier's study.

### 4.7 Brain malformations

A previously published study about prenatally diagnosed 1p36DS by Guterman and colleagues found 60% of brain abnormalities (Guterman et al., 2019). Interestingly, among brain malformations, Guterman and colleagues recorded 3/10 fetuses with ventriculomegaly and 2/10 fetuses with corpus callosum abnormalities. This is consistent with our observation with 66% of brain malformations, 13/62 patients with abnormal corpus callosum, and 12/62 patients with ventriculomegaly. This is also consistent with published data by Shimada and colleagues with 36% of enlargement of lateral ventricles and 24% of hypoplasia of corpus callosum. Interestingly, of 25 deletions of patients with corpus callosum defect (13 patients from the present study, 2 reported by Guterman et al. and 10 reported by Shimada et al.), 20 shared the loss of the *SKI* gene (Figure 1) (Guterman et al., 2019; Shimada et al., 2015). Baranek and colleagues have shown, in mice, that SKI, a transcriptional factor, maintains the neural stem cell pool and the specification of callosal neurons and that “the misspecified callosal neurons largely fail to form the corpus callosum” (Baranek et al., 2012). The *SKI* gene could be one of the genes that explain the abnormalities of the corpus callosum, along with *RERE* or also *MTOR* (Figure 4).

### 4.8 Microcephaly

Microcephaly was observed in 32/57 patients in our cohort and 60% (Heilstedt, Ballif, Howard, Lewis, et al., 2003) to 83% (Shimada et al., 2015) of patients in the literature. Microcephaly has also been described in Shprintzen‐Goldberg syndrome (Doyle et al., 2012). Forty‐five deletions from our cohort include the *SKI* gene, with 13 patients having microcephaly. We observed more patients with microcephaly in Group B (8/9) than in Group A, and in the literature, 5/6 patients with proximal deletions presented microcephaly, and the last one has a head circumference at the 5th centile. Therefore, microcephaly seems to be a frequent feature in proximal 1p36 deletions. It has been suggested that another gene: *RERE* (Arginine‐glutamic acid dipeptide repeats) may be responsible for microcephaly in 1p36DS. Indeed, Kim et al. observed a reduced brain size in *Rere* mutant mice (Kim et al., 2013). However, only two Group B deletions encompassed *RERE*. This suggests either a positional effect, indeed, deletions may be responsible for Topologically Associating Domains (TADs) disruption and chromatin disorganization leading to the deregulation of several genes or candidate genes for microcephaly as *CDC42* (Takenouchi et al., 2016), *SKI*, or *RERE* (Figures 4 and S1). Moreover, *MTOR* gain of function mutations have been described in a neurodevelopmental disorder associated with macrocephaly (Rodríguez‐García et al., 2019). *MTOR* is included in MOR3 and deleted in 10 patients, among these 10 patients, 8 presented microcephaly. Also, we can hypothesize that *MTOR* deletion may also be responsible for microcephaly (Figure 4).

### 4.9 Facial dysmorphism

In our post‐natal cohort, we observed more facial abnormalities, with 94% of patients reported with dysmorphism, than in the ACLF prenatal cohort (Guterman et al., 2019). This difference can be explained by the difficulty in identifying facial dysmorphism prenatally on ultrasound. Horizontal, straight eyebrows and enophtalmia were the most reported features, congruent with typical facial appearance described in the literature (Battaglia, 1993). Nineteen patients' photographs were analyzed using Face2gene (FDNA, Inc.), without clinical information and 1p36DS was not proposed at all as a diagnosis by this tool in four patients. Interestingly, among these four patients, three cases carried interstitial, proximal deletion, and two cases belonged to Group B (two others not classified). Patients with proximal deletions, described in the literature by Kang et al. and Rudnik‐Schoneborn et al. presented microcephaly (5/6), prominent forehead (3/6), bushy (2/6) and arched (3/6) eyebrows, long eyelashes (3/6), epicanthal folds (3/6), hypertelorism (3/6), and posteriorly rotated ears (3/6) (Kang et al., 2007; Rudnik‐Schöneborn et al., 2008). Typical deep‐set eyes were not reported in these patients. Thus, dysmorphism in patients with proximal deletions may be slightly different from classical distal deletions. Recently, Nolting et al. described “a new microdeletion syndrome at proximal 1p36 (1p36.13‐1p36.12)” in seven patients and determined a new SRO from 19,077,793 bp to 20,081,292 bp. These patients presented ID, speech delay, behavioral abnormalities, and congenital ptosis (Aagaard Nolting et al., 2020). Two patients in our cohort exhibit ptosis including one with a deletion (17175659‐22652664) encompassing the SRO described by Nolting.

### 4.10 Cardiac abnormalities

Guterman found 40% of cardiac malformations in the prenatal cohort (Guterman et al., 2019), similarly, we reported 47% of cardiovascular malformations or cardiomyopathy and we found more patients with cardiac abnormalities in Group A than in Group B. Only 2/11 patients in Group B presented cardiovascular abnormalities (Fallot tetralogy and high blood pressure). However, in the literature, 5/6 patients with proximal deletions presented structural heart defects and 2/6 cardiomyopathy (Rudnik‐Schöneborn et al., 2008). In the literature from 22 to 27% (Table 1) of patients (Battaglia et al., 2008; Shimada et al., 2015) presented cardiomyopathy and two critical regions for cardiomyopathy have been described one of which includes *PRKCZ*, *SKI*, and *PRDM16* (Zaveri et al., 2014). In our study, 21% (16/77) of patients presented cardiomyopathy. Of these 16 patients, deletions were mapped in 12 of them. All these 12 patients carried deletions belonging to Group A, and all encompassed the *PRDM16* gene (Figure 1). Indeed, mutations in *PRDM16* have been described in patients with cardiomyopathy and left ventricular non‐compaction (Arndt et al., 2013). However, about 20 patients in our study, with deletions encompassing *PRDM16* did not have any type of cardiac abnormalities suggesting a variable penetrance. Arndt and colleagues suggested a possible interaction between *PRDM16* and *SKI*. They coinjected subthreshold doses of *PRDM16* and *SKI* morpholinos in zebrafish and observed a reduced cardiac output (Arndt et al., 2013). Interestingly, three deletions from our cohort, encompassing *PRDM16* but no *SKI* did not present cardiomyopathy. Some other genes may be responsible for cardiomyopathy as *PDPN*, and *CASZ1* (Jordan et al., 2015). According to the literature, structural heart defect is observed in 43–71% of patients (Battaglia et al., 2008; Heilstedt, Ballif, Howard, Lewis, et al., 2003) (Table 1), and five critical regions have been described (Zaveri et al., 2014). We reported here 32% of patients with structural defects as VSD, PDA, and atrial septal defect, mostly in Group A. This is lower than that observed by Shimada et al. but, in the present study, data were only available for 77/86 patients so structural heart defects may be underestimated. It has been described that the haploinsufficiency of the *RERE* gene may be responsible for clinical signs recorded in 1p36 deletion, including heart defects notably ventricular septal defect (Figure 4) (Fregeau et al., 2016). The *ECE1* gene may also contribute to cardiac features. *ECE1* has been implicated in Hirschsprung disease, autonomic dysfunction, and cardiac defects. Hofstra et al. described a patient with Hirschsprung disease, ductus arteriosus, small subaortic ventricular septal defect, and small atrial septal defect and dysmorphism, carrier of a missense mutation in *ECE1* and they suggested that this variant may cause or contribute to the patient's phenotype (Hofstra et al., 1999).

## 5 CONCLUSION

Thanks to a collaboration with the ACLF we have collected data of 86 patients constituting the second‐largest cohort of 1p36DS patients in the literature to our knowledge. This study enables a continuum with prenatal published data. We estimated an average of, at least, 10 cases per year in France. 1p36DS seems to be much less frequent than 22q11.2 deletion syndrome and trisomy 21. We observed that 1p36DS may be a life‐threatening condition and can lead to early death in the most severe cases. Mapping and genotype–phenotype correlation allowed us to identify four critical regions responsible for ID and some phenotypic variability, particularly according to the deletion position. This study helps to refine the phenotype of 1p36DS, which is diagnosed more and more early, especially prenatally with the generalization of the CMA and more recently with genome analysis. A better knowledge of this syndrome allows improved management of patients, particularly in the detection of malformations, complications, and in the follow‐up of patients. Moreover, even in the face of characteristics specifically suggesting 1p36DS, a CMA would provide more information to families.

## AUTHOR CONTRIBUTIONS

Clémence Jacquin, Emilie Landais, Céline Poirsier, Alexandra Afenjar, Ahmad Akhavi, Nathalie Bednarek, Caroline Bénech, Adeline Bonnard, D. Bosquet, Lydie Burglen, Patrick Callier, Sandra Chantot‐Bastaraud, Christine Coubes, Bruno Delobel, Margaux Descharmes, Jean‐Michel Dupont, Vincent Gatinois, Nicoles Gruchy, Sarah Guterman, Abdelkader Heddar, Lucas Herissant, Delphine Heron, Bertrand Isidor, Pauline Jaeger, Guillaume Jouret, Boris Keren, Paul Kuentz, Cedric Le Caignec, Jonathan Levy, Nathalie Lopez, Zoe Manssens, Dominique Martin‐Coignard, Cyril Mignot, Chantal Missirian, Céline Pebrel‐Richard, Lucile Pinson, Jacques Puechberty, Sylvia Redon, Damien Sanlaville, Marta Spodenkiewicz, Anne‐Claude Tabet, Alain Verloes, Gaelle Vieville, Catherine Yardin, François Vialard, Martine Doco‐Fenzy clinically examined patients, provided clinical and genetics data, and/or performed genetic analysis. Clémence Jacquin designed the study and wrote the manuscript. Emilie Landais performed part of the data analysis. All authors read and approved the final version of the manuscript. Emilie Landais and Martine Doco‐Fenzy supervised the study, and critically reviewed the article.

## CONFLICTS OF INTEREST

The authors declare no conflict of interest.

## Supporting information


**Appendix S1:** Supporting Information.

**Table S4:** Summary of clinical features of all the patients

## Data Availability

The data that support the findings of this study are available on request from the corresponding author. The data are not publicly available due to privacy or ethical restrictions.
