# Supplementary material for: 1p36 deletion syndrome: Review and mapping with further characterization of the phenotype, a new cohort of 86 patients
Source: Am J Med Genet A. 2022 Nov 11;191(2):445–58. doi: 10.1002/ajmg.a.63041 (PMC10100125; doi:10.1002/ajmg.a.63041)
Supplement: Supplementary file 1 — Appendix S1: Supporting Information. [file AJMG-191-445-s002.docx]

**1p36 deletion syndrome: review and mapping with further characterization of the phenotype, a new cohort of 86 patients.** C. JACQUIN^1^  *et al*.

**Supplementary data**

**Table S1: Chromosomal microarray analysis results.**

| Centers | Patient number | Age | START ^a^ | END ^a^ | Additional aberration | Inheritance |
| --- | --- | --- | --- | --- | --- | --- |
| REIMS | del1 | 11 d | 564,424 | 12,035,607 |  | *dn* |
| REIMS | del2 | 2 m | 11,785,007 | 12,317,037 | arr[hg19] 1p34.2(42,073,164-42,477,540)x3,  1p33p31,3(46,811,413-67,254,184)x3 | NA |
| Caen | del3 | 22 m | 852,863 | 4,316,967 | arr[hg19] 16p13.11(15,048,751-16,276,115)x3 | *dn*  pat. |
| Caen | del4 | 31 y | 759,762 | 4,725,355 |  | *dn* |
| Caen | del5 | 2 y | 9,753,120 | 14,408,825 |  | *dn* |
| BREST | del6 | 15 y | 1 | 2,567,773 |  | *dn* |
| BREST | del7 | 2 y | 564,424 | 3,628,711 |  | *dn* |
| BREST | del8 | 8 y | 9,124,551 | 13,142,545 |  | *dn* |
| BREST | del9 | 3 y | 10,631,952 | 11,718,611 |  | *dn* |
| TRS | del10 | 12 y | 564,424 | 4,203,509 |  | *dn* |
| TRS | del11 | 2 y | 82,154 | 2,056,735 |  | *dn* |
| PSL | del12 | 15 y | 5,179,954 | 11,774,956 |  | *dn* |
| TRS | del13 |  | 82,154 | 5,310,258 |  | *dn* |
| Dijon | del14 | 9 m | 564,424 | 4,645,192 |  | *dn* |
| Dijon | del15 | 14 m | 7,792,675 | 15,955,670 |  | *dn* |
| Dijon | del16 | 5 m | 564-224 | 6,373,470 |  | *dn* |
| RDB | del17 | 18 m | 21,035,149 | 22,885,766 |  | *dn* |
| RDB | del18 | 16 y | 82,154 | 5,335,599 |  | NA |
| RDB | del19 | 20 y | 82,154 | 2,473,258 | arr[hg19] 9q34.2q34.3(136,682,482-141,066,491)x3 | NA |
| RDB | del20 | 30 m | 82,154 | 4,212,493 | arr[hg19] 3q29(194,854,022-197,838,262)x3 | NA |
| HCL | del21 | 1 y | 564,424 | 6,823,262 |  | *dn* |
| HCL | del22 | 20 y | 1 | 5,374,972 | mosaic |  |
| Marseille | del23 | 15 y | 746,608 | 5,710,280 |  | NA |
| Marseille | del24 | 15 m | 564,424 | 2,575,457 | arr[hg19] 1p36.32(2,704,715-4,128,515)x3 | *dn* |
| Marseille | del25 | 8 m | 1,613,750 | 5,334,068 |  | *dn* |
| Marseille | del26 | 8 m | 1,664,166 | 3,543,533 |  | NA |
| Marseille | del27 | 11 d | 746,608 | 3,739,423 |  | *dn* |
| Marseille | del28 | 22 y | 2,728,545 | 8,403,606 |  | *dn* |
| Marseille | del29 | 25 m | 7,836,776 | 14,933,994 |  | *dn* |
| Marseille | del30 | 4 y | 1,065,123 | 2,575,457 |  | *dn* |
| Marseille | del31 | 7 y | 948,462 | 2,780,510 |  | NA |
| Marseille | del32 | 4,5 y | 759,760 | 1,716,699 | arr[hg19] 1q42.3q44(235,551,308-249,212,610)x3 | *dn* |
| Marseille | del34 | 15 y | 801,556 | 2,575,457 |  | *dn* |
| Marseille | del35 | 11 y | 1,585,574  4,812,851 | 3,582,025  5,491,640 |  | *dn* |
| Marseille | del37 | 3 m | 1 | 5,053,721 |  | *dn* |
| Clermont-F | del40 | 7 m | 564,424 | 5,007,786 |  | *dn* |
| Clermont-F | del41 | 2y | 759,762 | 2,553,982 |  | *dn* |
| Limoges | del42 | 1 m | 835,601 | 10,159,771 |  | NA |
| Cochin | del43 | 2 d | 20,017,962 | 24,799,388 |  | *dn* |
| Limoges | del44 | 2 m | 759,762 | 3,070,509 |  | *dn* |
| REIMS | del45 | 2 m | 1,619,653 | 6,665,409 |  | *dn* |
| REIMS | del46 | 1 y | 746,608 | 6,130,435 | arr[hg19] 10q26,3(133,749,950-133,933,703)x1 | *dn* |
| REIMS | del47 | 3 y | 5,479,682 | 12,528,792 |  | NA |
| REIMS | del48 | 16 y | 564,424 | 3,324,561 |  | NA |
| REIMS | del49 | 46 m | 1 | 4,708,253 |  | NA |
| REIMS | del50 | 7 y | 1 | 4,708,253 |  | *dn* |
| HCL | del51 | 10 y | 564,405 | 4,958,499 | 6q27(A_16_P01641551->A_16_P01642188)x3 | NA |
| Cochin | del52 | 11 m | 27,918 | 6,502,218 |  | NA |
| BESANCON | del53 | 3 m | 564,424- | 2,704,774 | arr[hg19] 1p36.32p36.23(2712385-8022698)x1~2, 1p36.23(8308170-8712517)x1~2 | *dn* |
| TRS | del54 | 8 y | 5,528,517 | 17,366,871 |  | *dn* |
| TRS | del55 | 20 m | 82,154 | 6,580,397 |  | *dn* |
| Nantes | del56 | 8 d | 752,470 | 9,019,286 |  | NA |
| Marseille | del57 | 3 m | 779,727 | 3,518,608 |  | *dn* |
| Grenoble | del58 | 4 y | 17,175,659 | 22,652,664 |  | *dn* |
| TRS | del59 | 7,5 y | 82,154 | 6,414,084 |  | NA |
| TRS | del60 | 13 y | 82,154 | 5,073,658 |  | NA |
| Montpellier | del61 | 33 y | 849,466 | 2,057,167 |  | *dn* |
| Montpellier | del62 | 11 y | 2,785,042 | 10,753,137 | arr[hg19] (X)x1,(Y)x2 | NA |
| Montpellier | del63 | 11 d | 2,723,344 | 3,800,088 |  | mat. |
| Lille | del65 | 5 y | 10,732,710 | 14,059,339 |  | *dn* |
| Lille | del66 | 3 d | 759,762 | 5,696,605 |  | *dn* |
| Lille | del67 | 9 y | 759,762 | 2,553,982 |  | NA |
| Lille | del68 | 7 d | 14,408,766 | 23,660,218 |  | *dn* |
| Lille | del69 | 15 y | 3,354,713 | 8,343,813 |  | *dn* |
| Lille | del70 | 5 y | 759,759 | 5,411,803 | 6,XY,der(1),t(1;21)(p36.3;p11)pat | transloc. |
| Lille | del71 | 11 y | 5,578,090 | 6,939,206 |  | NA |
| Lille | del72 | 33 y | 564,405 | 3,390,238 |  | *dn* |

NA: not available; *dn: de novo ;* mat : maternally inherited; pat : paternally inherited; d : days; m : months; y : years; TRS : Armand-Trousseau Hospital; PSL: Pitié Salpêtrière - Charles Foix Hospital; RDB : Robert Debré Hospital; HCL : Civil Hospices of Lyon; Clermont-F: Clermont-Ferrand Hospital. ^A^: build GRCh37/hg19.

**Table S2: Clinical signs reported in our cohort.**

|  | **Total (n=86)** |
| --- | --- |
| **Developmental delay and/or Intellectual disability** | 75/75 |
| **Dysmorphism** | 73/78 |
| **Hypotonia** | 53/63 |
| **Epilepsy** | 46/68 |
| **Growth retardation pre and/or postnatal** | 45/57 |
| **Brain malformations** | 41/62 |
| Corpus callosum abnormalities | 13 |
| Ventriculomegaly | 12 |
| Cortical atrophy | 6 |
| White matter anomalies | 4 |
| Cerebral cysts | 4 |
| Myelination delay | 4 |
| Hemispheric atrophy | 3 |
| Pachygyria | 3 |
| Cortical dysplasia | 2 |
| Heterotopias | 2 |
| Polymicrogyria | 2 |
| **Cardiomyopathy / cardiovascular malformations** | 36/77 |
| Overriding aorta | 1 |
| Dilated cardiomyopathy | 6 |
| Hypertrophic cardiomyopathy | 2 |
| Atrial septal defect | 5 |
| Ventricular septal defect | 11 |
| Aortic coarctation | 1 |
| Dilation of the ascending aorta | 1 |
| Left ventricular dysfunction | 3 |
| Tetralogy of Fallot | 1 |
| High blood pressure | 2 |
| Pulmonary hypertension | 4 |
| Hypoplasia of the abdominal aorta | 2 |
| Heart failure | 1 |
| Left ventricular noncompaction | 7 |
| Patent ductus arteriosus | 9 |
| **Behavioral disorders** | 33/44 |
| **Hands abnormalities** | 33/51 |
| **Microcephaly** | 32/57 |
| **Eye / vision problems** | 28/40 |
| Strabismus | 14 |
| Hypermetropia | 7 |
| Nystagmus | 5 |
| Astigmatism | 5 |
| Cataract | 2 |
| Coloboma | 2 |
| Pupillary pager | 2 |
| Myopia | 1 |
| Tearing | 1 |
| Photophobia | 1 |
| Optic atrophy | 1 |
| Amblyopia | 1 |
| Retinal hemorrhage | 1 |
| Imperforation of the lacrimal ducts | 1 |
| **Feet abnormalities** | 25/46 |
| **Gastrointestinal anomalies** | 24/36 |
| Gastroesophageal reflux | 13 |
| Constipation | 9 |
| Liver abnormalities | 3 |
| Esophageal and gastric abnormalities | 2 |
| Umbilical Hernia | 2 |
| Duodenal atresia | 1 |
| Recto-vaginal fistula | 1 |
| Anal imperforation | 1 |
| Annular pancreas | 1 |
| **Spinal defects** | 24/40 |
| Scoliosis | 9 |
| Kyphosis | 6 |
| Spinal abnormalities | 3 |
| Sacrococcygeal dimple | 4 |
| Hyperlordosis | 3 |
| Gibbosity | 2 |
| Hemivertebra | 1 |
| Sacral fistula | 1 |
| Spina bifida | 1 |
| **Joint abnormalities** | 13/31 |
| Hyperlaxity | 6 |
| Joint limitations | 2 |
| Epiphysiolysis of the hips | 2 |
| Hip dysplasia | 1 |
| **Hearing loss** | 11/31 |
| **Skin abnormalities** | 11/31 |
| Angiomas | 4 |
| Eczema | 3 |
| Angiomas of the forehead | 2 |
| Spreading nipples | 2 |
| Pigmentation abnormalities | 1 |
| Umbilical nipples | 1 |
| Prominent nipples | 1 |
| Michelin tire baby | 1 |
| Stretch marks | 1 |
| **Limbs abnormalities** | 9/28 |
| **Lungs abnormalities** | 8/22 |
| Sleep Apnea Syndrome | 4 |
| Asthma | 2 |
| Bronchomalacia | 1 |
| Pulmonary hypoplasia | 1 |
| Laryngomalacia | 1 |
| **Abnormalities of the external genitalia** | 8/27 |
| Cryptorchidism | 6 |
| Phimosis | 2 |
| Oscillating testicles | 1 |
| Inguino scrotal hernia | 1 |
| Bicorn uterus | 1 |
| **Renal defects** | 6/27 |
| Pyelocaliceal / ureteral dilatation | 3 |
| Polycystic renal dysplasia | 2 |
| Renal lithiasis | 1 |
| Renal duplicity | 1 |
| **Others** |  |
| **Overweight** | 9 |
| Hypertrichosis | 7 |
| Early puberty | 5 |
| Hypertonia/pyramidal syndrome | 5 |
| Hypothyroidism | 4 |
| Hematologic disorders | 2 |

**Table S3: Focus on patients with regression of acquisitions**

| Patient number | START | END | Epilepsy | Brain malformations |
| --- | --- | --- | --- | --- |
| del3 | 852,863 | 4,316,967 | epileptic spasms, suspicion of West syndrome | Corpus callosum hypoplasia, myelination delay |
| del22 | 1 | 5,374,972 | epileptic encephalopathy, generalized seizures | Right-hemispheric atrophy, right temporo-fronto-parietal pachygyria |
| del48 | 564,424 | 3,324,561 | West syndrome, focal seizures with a right central origin, migrating to the left, hypsarrhythmia, and epileptic spasms | enlargement of the peri-cerebral spaces |
| del70 | 759,759 | 5,411,803 | generalized tonic-clonic epilepsy, suspicion of West syndrome | diffuse cerebral atrophy, moderate enlargement of basal cisterns, moderate ventriculomegaly, White matter anomalies |

**Table S4: Summary of clinical features of all the patients**

NA : not available; + : presence; - : absence; F : female; M : male; Y : yes; N : no; DD : developmental delay; ID : intellectual disability; GR : growth retardation; CMP : cardiomyopathy; CVM : cardiovascular malformations; P : early-onset puberty; H : hypertrichosis; T : hypothyroidism; F : Large fontanel; Ov Ao : Overriding aorta; Ao Co : Aortic coarctation; DAA : Dilation of the ascending aorta; T4F : Tetralogy of Fallot; HF: Heart failure. Patients del33, del36, del39, del64, and del73 were excluded from the analysis.

**Provided in a separate file.**


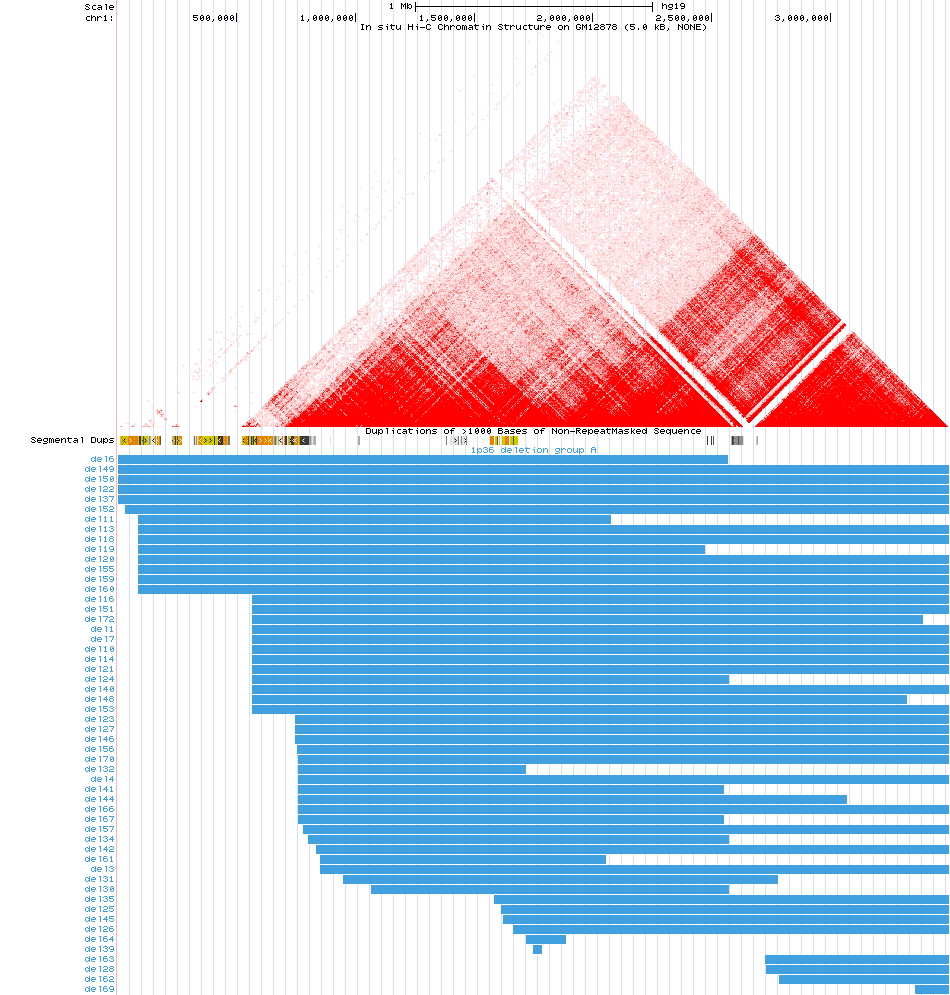


**Figure S1: Mapping of deletions diagnosed by microarray using UCSC Genome Browser (build GRCh37/hg19), segmental duplications and Topologically Associating Domains (TADs) analysis within the 1p36.11p36.33 region.** Blue bars represent group A deletions (n= 56 patients), TADs predicted by Hi-C analysis in GM12878 cell line (Rao *et al.* 2014.


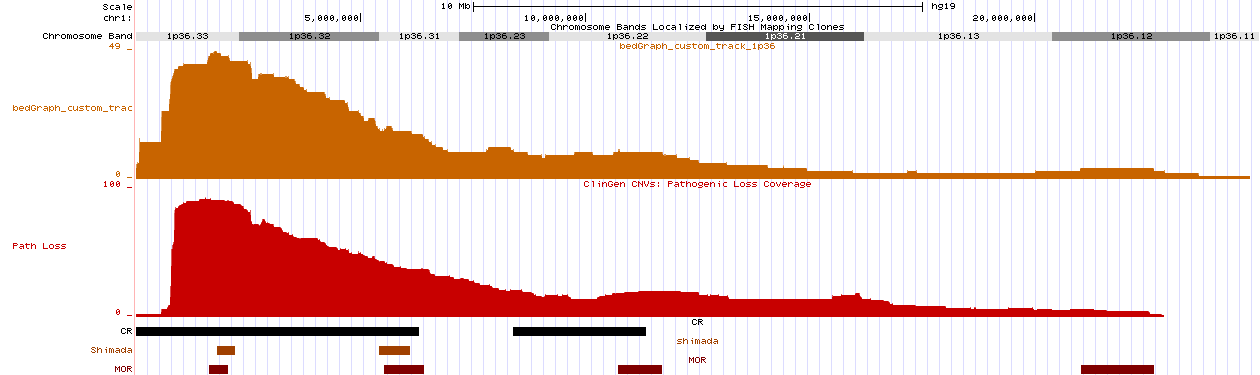


**Figure S2: Graphical representation of all deletions in our cohort using UCSC Genome Browser (build GRCh37/hg19).** The orange graph represents all deletions in our cohort plotted on a graph, the red graph represents “ClinGen CNV pathologic loss coverage”, the horizontal black bars represent distal and proximal critical regions described in the literature, the horizontal orange bars critical regions described by Shimada et al., the horizontal red bars and red translucent vertical lines represent the proposed four minimal overlapping regions (MOR) : MOR1 : 1,619,654-2,057,167; MOR2 : 5,528,518-6,414,084; MOR3 : 10,732,711-11,718,611; MOR4 : 21,035,150-22,652,664.
